# Assessment of Outcomes of Endonasal Dacryocystorhinostomy in a Tertiary Referral Center

**DOI:** 10.7759/cureus.105621

**Published:** 2026-03-21

**Authors:** Shreya Kovuru, Usha G, Bosco Suriya Luke Rathnakumar, S.M. Azeem Mohiyuddin

**Affiliations:** 1 Otorhinolaryngology - Head and Neck Surgery, Sri Devaraj Urs Academy of Higher Education and Research, Kolar, IND; 2 Otorhinolaryngology/Rhinology and Skull Base Surgery, Sri Devaraj Urs Medical College, Kolar, IND; 3 Otorhinolaryngology - Head and Neck Surgery, Sri Devaraj Urs Medical College, Kolar, IND

**Keywords:** chronic dacryocystitis (cd), deviated nasal septum (dns), diabetes mellitus (dm), endoscopic dacryocystorhinostomy (endo-dcr), lacrimal sac syringing (lss), odds ratio (or)

## Abstract

Background

Chronic dacryocystitis due to nasolacrimal duct (NLD) obstruction is a common cause of epiphora and lacrimal abscess. Endonasal dacryocystorhinostomy (Endo-DCR) offers a scarless approach with outcomes comparable to those of external dacryocystorhinostomy when performed with appropriate technique and adequate follow-up.

Objectives

To evaluate the surgical outcomes of Endo-DCR and to identify risk factors associated with postoperative complications and surgical failure.

Methods

A retrospective observational study was conducted on 60 patients (18-70 years) with chronic dacryocystitis who underwent Endo-DCR. Follow-up data were collected at the end of one week, one month, three months, and six months following surgery. Success was defined as symptomatic resolution of epiphora with patent lacrimal syringing and endoscopic stomal patency at the end of six months. Fisher’s exact test, independent t-test, and univariate binary logistic regression were used. P < 0.05 was considered significant.

Results

At the end of six months, patency on lacrimal sac syringing and stomal patency on endoscopy were seen in 78.3% (n = 47/60) of patients. Immediate complications were generally mild, including nasal bleeding in 3.3% (n = 2/60) and lid edema in 8.3% (5/60). Delayed complications included granulation tissue (8.3%, n = 5/60), synechiae (6.7%, n = 4/60), and stomal closure (6.7%, n = 4/60). Failure was associated with diabetes mellitus (DM) and deviated nasal septum (both p < 0.05). Deviated nasal septum (OR: 2.52) and DM (OR: 3.05) are predominant factors predicting failure. An additional insight from this study is the pattern seen with multiple concurrent risk factors. Combinations such as DM with smoking and DM with deviated nasal septum appeared to cluster among failures, suggesting a potential synergistic risk profile rather than purely additive effects.

Conclusion

Primary Endo-DCR achieved a high success rate in this tertiary center cohort. Deviated nasal septum and DM were associated with failure. Highlighting the importance of risk stratification, optimization of systemic disease, and concurrent management of nasal pathology to improve outcomes.

## Introduction

Epiphora is a frequent complaint in ophthalmology and otorhinolaryngology practice and is commonly caused by obstruction of the lacrimal drainage system. Nasolacrimal duct (NLD) obstruction accounted for 31.8% of all chronic epiphora and 67.6% of all lacrimal drainage pathway obstructions [1]. Acquired NLD obstruction predisposes to chronic dacryocystitis, typically presenting with persistent tearing, mucopurulent discharge, and recurrent infection. Epidemiologic work suggests a higher burden in middle-aged and older adults and a female predominance, attributed in part to anatomical differences in the nasolacrimal canal and mucosal factors [2,3].

Endonasal dacryocystorhinostomy (Endo-DCR) is the definitive treatment for symptomatic NLD obstruction, creating a bypass between the lacrimal sac and the nasal cavity. It avoids a cutaneous incision and facilitates direct visualization of the rhinostomy site, with success rates comparable to external approaches when meticulous mucosal handling and postoperative care are ensured [4-8].

Despite generally favorable outcomes, failure still occurs, most often due to ostium stenosis/closure from granulation tissue and synechiae. Systemic comorbidities such as diabetes mellitus (DM) can impair wound healing and amplify fibrosis, potentially increasing restenosis risk [7].

Local nasal pathology, such as a deviated nasal septum (DNS), may also compromise surgical access and postoperative healing if not addressed [8].

While several studies have reported outcomes of Endo-DCR in different settings [4-9], there remains limited evidence correlating specific patient- and disease-related risk factors with outcomes in rural tertiary referral populations. This study, therefore, evaluated six‑month outcomes of Endo-DCR and explored predictors of surgical failure.

Aim

To assess the outcomes of Endo‑DCR performed for chronic dacryocystitis in a tertiary referral center.

Objectives

To identify risk factors associated with Endo-DCR failure in the study population. To estimate postoperative complications following Endo‑DCR. To correlate identified risk factors with postoperative complications and final surgical outcome at six months.

## Materials and methods

Inclusion criteria

The inclusion criteria included adults aged 18-70 years with chronic dacryocystitis who underwent Endo-DCR.

Exclusion criteria

The exclusion criteria included patients with revision Endo-DCR, extensive facial trauma, prior major surgery involving the lateral nasal wall, lacrimal abscess, and patients with a tendency to develop keloids.

Methodology

A retrospective review of hospital records for all patients admitted with a diagnosis of chronic dacryocystitis admitted to the Department of Otorhinolaryngology - Head and Neck Surgery at R.L. Jalappa Hospital, affiliated to Sri Devaraj Urs Academy of Higher Education and Research, a tertiary referral center, was conducted between 1st January 2020 to 30th June 2025. A total of 76 patient records were initially identified. Records were screened for eligibility, and patients were excluded if they had incomplete documentation (n = 2), had absconded during treatment (n = 4), or had been discharged against medical advice (n = 10). After applying these exclusion criteria, a total of 60 patients with complete and detailed clinical documentation were included in the final analysis.

Medical records were reviewed for demographics, clinical presentation, laterality, and associated risk factors (smoking, diabetes mellitus, nasal pathology, DNS, chronic rhinosinusitis (CRS), nasal polyposis, and turbinate hypertrophy).

All patients underwent Endo-DCR by two surgeons of equal expertise who followed the same surgical steps, which are as follows: under endoscopic visualization, the lateral nasal wall mucosa overlying the lacrimal sac region was elevated, bony exposure was achieved, and the lacrimal sac was marsupialized to create a wide rhinostomy. Meticulous hemostasis, removal of obstructing bone, and preservation of mucosal flaps were emphasized to reduce granulation and restenosis risk. Concomitant correction of nasal pathology, such as septal deviation, was performed when required for access and postoperative care. Medical records were reviewed for intraoperative findings and postoperative complications.

Follow-up and assessments

Patients were evaluated on postoperative days 7, 30, 90, and 180. The patients were evaluated for symptom status (epiphora), lacrimal syringing findings (free flow vs. regurgitation), and diagnostic nasal endoscopy findings (stomal patency, presence of granulation tissue, and synechiae).

Outcome measures

The primary outcome was resolution of epiphora. Secondary outcomes were functional patency on lacrimal syringing and anatomical patency on visualization of stoma on nasal endoscopy, and complication rates.

Definition of success

Success at 180 days required (i) complete resolution of epiphora, (ii) free flow of fluid on lacrimal sac syringing (LSS), and (iii) stomal patency on nasal endoscopy.

Statistical analysis

Descriptive statistics are reported as mean ± SD or frequency (%). Fisher’s exact test assessed categorical associations, and an independent t-test compared the age between outcome groups. A p-value of <0.05 was considered statistically significant.

## Results

Table 1 summarizes the demographic profile of the study population (n = 60). The majority of patients were in the age group of 31-50 years (46.7%, n = 28/60), with a mean age of 45.8 ± 12.6 years, and there was a female predominance (60%, n = 36/60). Overall, the cohort reflects a typical middle-aged population affected by NLD obstruction.

**Table 1:** Demographic distribution (n = 60).

Variable	Number (n)	Percentage (%)
Age (years)		
18-30	12	20.0
31-50	28	46.7
51-70	20	33.3
Mean age ± SD	45.8 ± 12.6	—
Gender		
Female	36	60.0
Male	24	40.0

Table 2 describes clinical presentation and laterality. Most patients had unilateral disease (93.3%, n = 56/60), with the right side slightly more commonly affected than the left. Epiphora alone was the most frequent symptom (60%, n = 36/60), while epiphora with discharge was present in 40% (n = 24/60).

**Table 2:** Clinical characteristics (n = 60).

Variable	Number (n)	Percentage (%)
Laterality		
Unilateral	56	93.3
Bilateral	4	6.7
Side involved (unilateral cases)		
Right	30	53.6
Left	26	46.4
Presenting symptom		
Epiphora	36	60%
Discharge with epiphora	24	40%

Table 3 lists the distribution of associated risk factors among cases. Diabetes mellitus (21.7%, n = 13/60) was the most frequent systemic comorbidity, while DNS (13.3%, n = 8/60) and smoking (13.3%, n = 8/60) were the most common local/lifestyle factors; CRS was also noted in a smaller proportion. Nearly 45% (n = 27/60) had no identifiable risk factor, indicating that a substantial number of NLD obstruction cases occurred without obvious predisposing conditions.

**Table 3:** Risk factors in the cohort (n = 60).

Risk factor	Number (n)	Percentage (%)
Diabetes mellitus	13	21.7%
Deviated nasal septum	8	13.3%
Chronic rhinosinusitis	4	6.7%
Smoking	8	13.3%
No identifiable risk factor	27	45%

Table 4 outlines postoperative complications, dividing them into immediate and delayed events. Immediate complications occurred in 7/60 cases, mainly lid edema and nasal bleeding, which were self-limited. Delayed complications were noted in 13/60 cases, most commonly granulation tissue (8.3%, n = 5/60), synechiae (6.7%, n = 4/60), and stomal closure (6.7%, n = 4/60), suggesting that late healing-related changes were the primary contributors to adverse outcomes.

**Table 4:** Postoperative complications.

Immediate postoperative complications	Frequency	Delayed postoperative complications	Frequency
Nasal bleeding	2	Granulation tissue formation	5
Lid edema	5	Synechiae	4
		Stomal closure	4
Total	7/60	Total	13/60

Tables 5, 6 represent functional patency on syringing over time. Free flow was observed in all patients at day seven (100%, n = 60/60), but gradually decreased to 78.3% (n = 47/60) by day 180, with a progressive increase in regurgitation from the same punctum reaching 21.7% (n = 13/60) at six months. This trend suggests that functional obstruction typically develops later, likely related to postoperative scarring or restenosis. A patent stoma was seen in 100% (n = 60/60) at day seven, reducing to 78.3% (n = 47/60) at day 180, while a closed stoma was documented in 6.7% (n = 4/60) at six months. The table also demonstrates evolving local findings over follow-up, with granulation tissue (8.3%, n = 5/60) and synechiae (6.7%, n = 4/60) appearing more commonly later, supporting their role in restenosis and failure.

**Table 5:** Lacrimal syringing findings at follow-up (n = 60).

Syringing result	Number at 7^th^ day	Number at 30^th^ day	Number at 90^th^ day	Number at 180^th^ day
Free flow of fluid	60 (100%)	54 (90%)	51 (85%)	47 (78.3%)
Regurgitation from the same punctum	0	06 (10%)	09 (15%)	13 (21.7%)

**Table 6:** Diagnostic nasal endoscopy findings at follow-up (n = 60).

Endoscopic finding	Number at 7^th^ day	Number at 30^th^ day	Number at 90^th^ day	Number at 180^th^ day
Patent stoma	60 (100%)	54 (90%)	51 (85%)	47 (78.3%)
Granulation tissue	0	5 (8.3%)	5 (8.3%)	5 (8.3%)
Synechiae	0	1 (1.7%)	4 (6.7%)	4 (6.7%)
Closed stoma	0	0	0	4 (6.7%)

Table 7 summarizes the overall surgical outcome at the end of follow-up. At six months, 47 patients (78.3%, n = 47/60) achieved success, while 13 patients (21.6%, n = 13/60) were classified as failures. These results reflect the combined functional and anatomical criteria used to define the final outcome in the study.

**Table 7:** Final surgical outcome at six months (n = 60).

Outcome	Number (n)	Percentage (%)
Successful outcome	47	78.3
Failure	13	21.6

Table 8 evaluates the association of selected risk factors with surgical outcome using Fisher’s exact test and reports corresponding odds ratios. The table suggests higher failure odds in patients with diabetes mellitus and DNS, while associations with CRS and smoking appear less consistent, likely influenced by smaller subgroup sizes.

**Table 8:** Association between risk factors and outcomes.

Risk factor	Present/absent	Success	Failure	Odds ratio	Fisher’s exact p-value
Diabetes mellitus (n = 13)	Present	8	5	3.05	0.130
	Absent	39	8		
Deviated nasal septum (n = 8)	Present	5	3	2.52	0.353
	Absent	42	10		
Chronic rhinosinusitis (n = 4)	Present	2	2	4.09	0.202
	Absent	45	11		
Smoking (n = 8)	Present	5	3	2.52	0.353
	Absent	42	10		

Table 9 focuses on patients with multiple concurrent risk factors and their outcomes. Failures were particularly prominent in combinations involving diabetes, especially diabetes with smoking (six failures out of 60) and diabetes with DNS (four failures out of 60), indicating that coexisting systemic and local risk factors may compound the likelihood of restenosis and surgical failure.

**Table 9:** Predictors of failure. DNS: deviated nasal septum; CRS: chronic rhinosinusitis.

Risk factor combination	Success	Failure	OR
Diabetes + DNS	1	4	20.44
DNS + CRS	0	2	20.65
DNS + smoking	0	1	11.40
Diabetes + smoking	1	6	39.43

**Figure 1:**
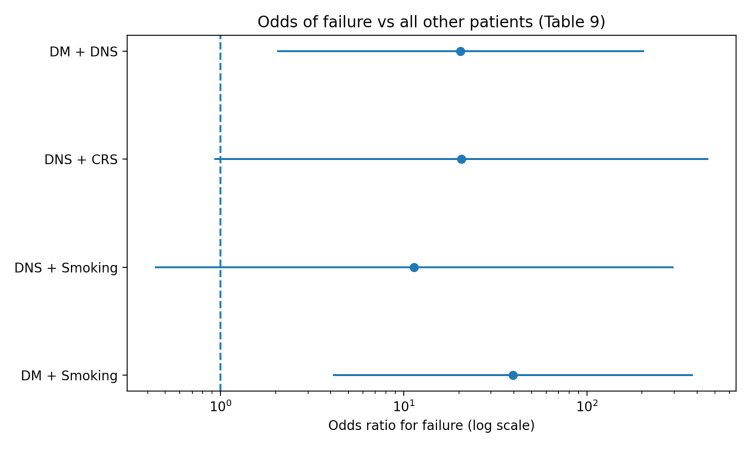
Forest plot of failure odds ratios for combined risk factors. DM: diabetes mellitus; DNS: deviated nasal septum; CRS: chronic rhinosinusitis.

The forest plot in Figure 1 summarizes the odds of surgical failure for patients with multiple concurrent risk factor combinations compared with the remainder of the cohort. The plot displays the odds ratio (OR) with 95% confidence intervals on a logarithmic scale, with the vertical reference line at OR = 1 indicating no difference in odds. All combinations showed ORs greater than 1, suggesting higher failure likelihood, with the largest effect estimate observed for DM with smoking, followed by DM with DNS. The confidence intervals are wide, reflecting the small subgroup sizes for these combinations.

## Discussion

Dacryocystitis is frequently encountered in patients presenting with epiphora, and LSS helps confirm NLD obstruction. Although external dacryocystorhinostomy (DCR) was once regarded as superior to the endoscopic approach, refinements in endoscopic DCR in recent years have markedly improved outcomes. These advances reflect progress in technology, better endoscopic instrumentation, and increasing surgical expertise.

In our cohort, patients ranged from 18 to 70 years (Table 1). Most participants fell in the age bracket of 31-50 years (28/60, 46.7%), followed by 51-70 years (20/60, 33.3%) and 18-30 years (12/60, 20.0%). The mean age was 45.8 ± 12.6 years, indicating a predominance of middle-aged patients, which is comparable to findings reported in other studies [1].

Females constituted the majority of cases (36/60, 60.0%; Table 1), while males accounted for 24/60 (40.0%), reflecting a female preponderance (approximately 1.5:1). This trend has been reported by other authors as well, and is often attributed to anatomical differences such as a relatively narrower bony nasolacrimal canal in females [10]. Hormonal influences, including menstrual and peri-menopausal fluctuations, have also been proposed as contributing factors to the higher prevalence in women, especially in the middle-aged group [11].

Regarding laterality, the disease was predominantly unilateral in 56/60 patients (93.3%), while 4/60 (6.7%) had bilateral involvement. Among the unilateral cases, the right side was affected in 30/56 (53.6%) and the left side in 26/56 (46.4%; Table 2).

In our sample population, several patient- and nasal-related risk factors were identified that may influence Endo-DCR outcomes (Table 3). A subset of patients had systemic comorbidities, most notably DM. DM was associated with failure and remained a significant predictor in our analysis, even when present in association with other risk factors such as DNS (OR: 3.05; Tables 8, 9). This is biologically plausible because hyperglycemia impairs neutrophil function, collagen remodeling, and epithelialization, promoting fibrotic healing and restenosis [7]. In patients who developed granulations at the stoma, topical steroidal spray (fluticasone furoate) was started and continued till the granulation tissue subsided. Optimizing preoperative glycemic control and ensuring appropriate postoperative care in terms of early identification and mitigation of the granulations in high‑risk individuals may therefore improve outcomes.

Smoking was another relevant systemic factor in a smaller proportion of cases and is known to impair mucociliary clearance and wound repair, potentially increasing crusting, granulation tissue, and adhesion formation.

DNS was the second most common contributing factor, also associated with failure after DM. Septal deviation can reduce surgical access, limit osteotomy size, and contribute to postoperative synechiae by narrowing the nasal corridor [8]. Although patients underwent surgical correction of the deviated septum in the same sitting, the surgical trauma may exacerbate synechiae formation in the postoperative period, thereby compromising stomal visualization.

In addition, CRS, when present, may contribute to a persistently inflamed intranasal environment that favors edema, granulation, and cicatricial narrowing of the stoma. Notably, some patients exhibited multiple concurrent risk factors (e.g., diabetes with smoking or diabetes with DNS), and these combinations may act synergistically by combining impaired systemic healing with unfavorable local anatomy, thereby increasing the likelihood of postoperative ostial stenosis and surgical failure.

Table 5 demonstrates a progressive decline in functional patency on lacrimal syringing over the follow-up period. While all 60 patients (100%, n = 60/60) showed free flow on day seven, this reduced to 54 (90%, n = 54/60) by day 30 and 51 (85%, n = 51/60) by day 90. By six months (day 180), free flow was present in 47 patients (78.3%, n = 47/60), whereas regurgitation from the same punctum increased from 0% at day seven to 10% at day 30, 15% (n = 3/60) at day 90, and 21.7% (n = 13/60) at day 180. Overall, the table highlights that most failures appear as delayed functional obstruction, supporting the concept that postoperative healing changes (granulation, adhesions, and scarring) play a major role in late reduction of patency.

Table 6 outlines the anatomical evolution of the rhinostomy site on endoscopic follow-up. A patent stoma was seen in all patients (n = 60, 100%) at day seven, decreasing to 54/60 (90%) at day 30 and 51/60 (85%) at day 90. At six months, 47/60 patients (78.3%) continued to show a patent stoma, while a closed stoma was documented in 4/60 patients (6.7%). Notably, endoscopy also identified the mechanical contributors to failure: granulation tissue was absent at day seven but appeared by day 30 in 5/60 patients (8.3%) and persisted at the same frequency through day 90 and day 180; synechiae increased over time from 1/60 patients (1.7%) at day 30 to 4/60 patients (6.7%) at day 90 and day 180. These findings emphasize that restenosis is typically driven by progressive intranasal scarring processes, and they support the need for structured postoperative endoscopic surveillance and timely management of granulation tissue and adhesions to preserve long-term stoma patency.

In this retrospective case series, primary Endo‑DCR achieved an overall six‑month success rate of 78.3% (n = 47/60), with concordant functional (syringing) and anatomical (endoscopic) patency rates (Table 7). These results align with published outcomes in tertiary settings, where success commonly ranges from the mid‑80% to low‑90% depending on case mix, definition of success, and follow-up duration [4-8].

Failure in the present cohort was largely attributable to stomal closure, consistent with the established pathophysiology of postoperative restenosis. The progressive rise in granulation tissue and synechiae on endoscopy across follow‑up underscores the importance of meticulous mucosal technique, adequate osteotomy size, and structured postoperative endoscopic care [4,7,8].

An additional insight from this study is the pattern seen with multiple concurrent risk factors (Tables 8, 9). Although the subgroup counts are small, combinations such as DM with smoking and DM with DNS appeared to cluster among failures, suggesting a potential synergistic risk profile rather than purely additive effects. Conceptually, this is plausible: systemic impairment in wound repair (DM ± smoking) combined with a mechanically constrained nasal corridor (DNS) may predispose to granulation, synechiae, and eventual stomal closure. Because these combinations include very few cases, formal inference is limited, and estimates can become unstable (particularly when a subgroup has no successes). Nevertheless, the finding is clinically useful as a signal: patients with two or more concurrent risk factors may warrant “enhanced surveillance” follow-up schedules, a lower threshold for early intervention, and more intensive counseling about adherence.

Strengths

Strengths of the study include the use of explicit, clinically meaningful success criteria incorporating symptoms, syringing, and endoscopic assessment, along with predefined follow-up time points.

Limitations

The retrospective design introduces risks of incomplete documentation and unmeasured confounding (e.g., exact osteotomy size, subtle differences in technique, and postoperative medication adherence). The sample size is modest, and the number of failures is limited, restricting the robustness of regression analyses and limiting the ability to perform reliable multivariable modeling with multiple predictors. Subgroup analyses of combined risk factors (Table 9) are therefore best framed as exploratory. Future work would benefit from a prospective design, standardized operative documentation (including osteotomy dimensions and intraoperative findings), structured postoperative protocols, and a larger cohort to enable adequately powered multivariable analysis and more definitive assessment of risk-factor interactions.

## Conclusions

Within the limitations of a retrospective design, Endo-DCR was an effective treatment for NLD obstruction in our study population, producing good short-term outcomes at six months. Failures were largely attributable to stomal restenosis related to granulation and synechiae, emphasizing that postoperative care is a critical determinant of success. DM and DNS emerged as clinically relevant predictors of poorer outcomes, and combined risk factor profiles appeared to further increase failure likelihood. Therefore, careful preoperative assessment of nasal anatomy, optimization of systemic comorbidities, and proactive endoscopic postoperative care, particularly in high-risk patients, may help reduce restenosis and improve surgical success.
